# Vascular Aldosterone Production at the Pre-Diabetic Stage of Young Otsuka Long-Evans Tokushima Fatty (OLETF) Rats, Compared with Long-Evans Tokushima Otsuka (LETO) Rats

**DOI:** 10.3390/molecules181215636

**Published:** 2013-12-13

**Authors:** Yoko Matsuzawa, Sachiko Suematsu, Jun Saito, Masao Omura, Tetsuo Nishikawa

**Affiliations:** Endocrinology and Diabetes Center, Yokohama Rosai Hospital, 3211 Kozukue-cho, Kohoku-ku, Yokohama 222-0036, Japan

**Keywords:** CYP11B2, aldosterone, diabetes, LETO, OLETF, aortic smooth muscle cells

## Abstract

We examined the ability of aortic smooth muscle cells (AoSMC) prepared from spontaneously diabetic rats to produce aldosterone (Aldo) and the regulatory mechanism that controls their Aldo production. AoSMC of 6 week-old Long-Evans Tokushima Otsuka (LETO: the control group) and 6 week-old Otsuka Long-Evans Tokushima Fatty (OLETF: the type 2 diabetes group) rats were used in the present experiments. CYP11B2 (Aldo synthetase) mRNA expression was detected in both the LETO and OLETF AoSMC. Basal Aldo production was significantly greater (4–5 fold higher) in the OLETF AoSMC culture medium than in the LETO AoSMC culture medium. When AoSMC were co-incubated with high-density lipoproteins (HDL), supplying cholesterol as a substrate for steroidogenesis in rats, angiotensin II (AII) significantly increased greater Aldo production in the OLETF AoSMC than in the LETO AoSMC. The present data suggested that future onset of diabetic vascular dysfunction is partly caused by excess Aldo production by AoSMC in young OLETF rats. Concomitant stimulation by HDL and AII resulted in elevated Aldo production in the OLETF and the LETO AoSMC, and also demonstrated that AII-induced Aldo production is greatly enhanced by HDL in OLETF, rather than in LETO. In conclusion, our data clearly demonstrated that Aldo production in the OLETF AoSMC was significantly higher than in the LETO AoSMC, suggesting possible future onset of vascular dysfunction in diabetes, induced by local Aldo production in the AoSMC.

## 1. Introduction

Recent studies have shown that aldosterone is produced by various extra-adrenal tissues, including vascular smooth muscle cells [1,2,3], cardiac muscle cells [4], glomerular mesangial cells [5], the kidneys [6], and the brain [7]. Thus, it was suggested that the aldosterone/mineralocorticoid receptor (MR) signaling pathway represents an ideal candidate for the pathway linking the early promoters of diabetes, especially overnutrition and obesity, with vascular insulin resistance [8], although the physiological relevance of local aldosterone production remains unclear. Moreover, an increasing body of evidence has underlined the importance of aldosterone in cardiovascular complications associated with metabolic syndrome, such as arterial remodeling and endothelial dysfunction, and hence, MR blockade is increasingly being used as a form of evidence-based therapy for many forms of cardiovascular disease, including hypertension, heart failure, chronic kidney disease, and diabetes mellitus (DM) [9].

We previously demonstrated that human primary mesangial cells express CYP11B2 mRNA, and the incubation of these cells with high levels of glucose significantly increased their aldosterone production compared with when they were incubated with physiological levels of glucose [10]. In addition, the upregulation of the systemic renin-angiotensin-aldosterone system is one of the most important pathological changes associated with the progression of diabetic nephropathy [11].

Otsuka Long-Evans Tokushima Fatty (OLETF) rats exhibit phenotypes that resemble those of humans with type 2 DM, for example, they display a chronic disease course involving mild obesity and possess multiple recessive genes for the induction of DM [12]. The associated control strain, LETO, appears to share some diabetogenic genes with OLETF rats [12]. It is also very interesting that vascular damage, including endothelial dysfunction, was reported to exist at 13 week-old in OLETF rats [13]. Previous studies have detected vascular aldosterone formation in hypertensive rats [1,2,3]; however, no such findings have been obtained for diabetic rat models.

Therefore, to elucidate the mechanism responsible for vascular aldosterone synthesis in various pathological cardiovascular conditions we examined the ability of aortic smooth muscle cells (AoSMC) prepared from spontaneously diabetic OLETF rats to produce aldosterone and the regulatory mechanism that controls their aldosterone production. Especially, we attempted to investigate the characteristics and nature of vascular aldosterone synthesis in young OLETF rats at the pre-diabetic stage in order to clarify the future onset of vascular complications in diabetes.

## 2. Results and Discussion

### 2.1. CYP11B2 mRNA Expression in Aortic SMC

**Figure 1:**
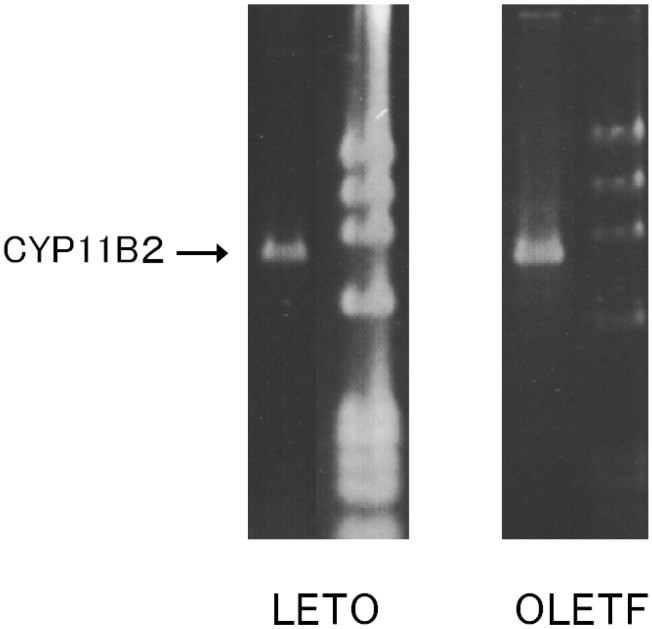
RT-PCR analysis of the CYP11B2 mRNA expression of AoSMC prepared from OLETF diabetic rats and control LETO rats.The mRNA of the indicated molecules was reverse-transcribed, and the resultant cDNA was amplified with RT-PCR. Ø × 174 fragments that had been digested with *Hae* III were added to the right lane as a molecular weight marker.

The AoSMC of the LETO and OLETF rats were confirmed to express CYP11B2 mRNA, as shown in Figure 1.

### 2.2. Pregnenolone Production

**Figure 2:**
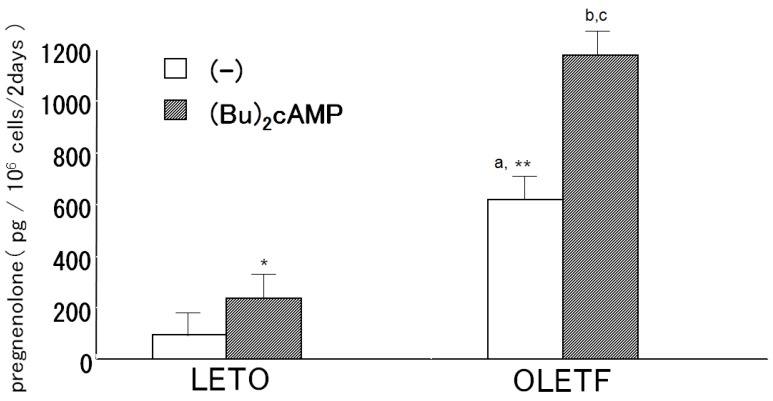
Pregnenolone production by rat AoSMC. The cells were cultured with/without (Bu)2cAMP for 2 h and then incubated with 30 μM trilostane, which is an antagonist of 3βHSD. Pregnenolone concentrations were assessed using a specific radioimmunoassay. Results are expressed as the mean ± S.E.M. * *p* < 0.05 *vs.* control (no additives); ** *p* < 0.001 *vs.* control of LETO (no additives). ^a^ *p* < 0.01 *vs.* +(Bu)2cAMP of LETO, ^b^ *p* < 0.01 *vs.* control of OLETF (no additives), ^c^ *p* < 0.001 *vs.* +(Bu)2cAMP of LETO.

The culture medium of the OLETF AoSMC exhibited significantly greater basal pregnenolone production than the LETO AoSMC culture medium (Figure 2). When the OLETF and LETO AoSMC were incubated with 1.0 mM (Bu)2cAMP in the presence of 30 μM trilostane for 2 h, significantly greater pregnenolone production was observed in the OLETF AoSMC culture medium than in the LETO AoSMC culture medium (Figure 2).

### 2.3. Aldosterone Production

#### 2.3.1. Effects of Angiotensin II and Losartan

**Figure 3:**
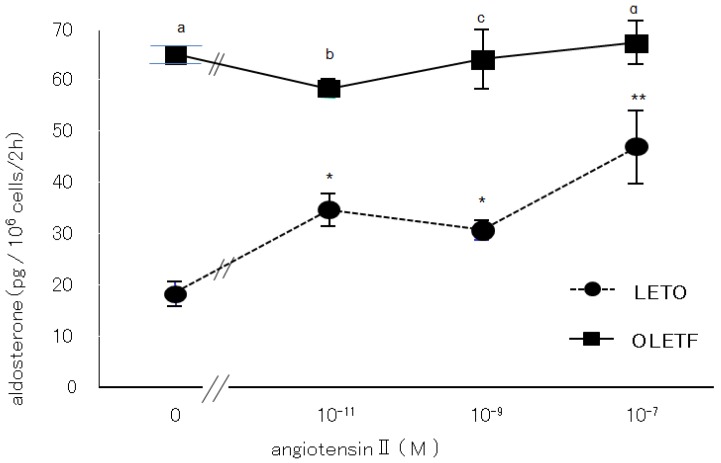
Effects of angiotensin II on aldosterone synthesis. The aldosterone production of rat AoSMC that had been cultured with or without angiotensin II for 2 h was determined as described in the text. The vertical bars represent the S.E.M. of the mean. * *p* < 0.01 *vs.* control (no additives); ** *p* < 0.001 *vs.* control (no additives). ^a^ *p* < 0.001 *vs.* control of LETO (no additives & +10^−9^ M AII ), ^b^ *p* < 0.01 *vs.* +10^−11^ M AII of LETO, ^c^ *p* < 0.001 *vs.* +10^−9^ M AII of LETO, ^d^ *p* < 0.05 *vs.* +10^−7^ M AII of LETO.

**Figure 4:**
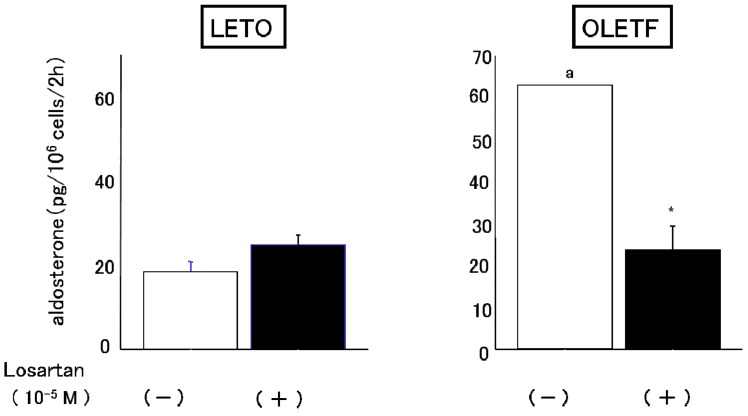
Effect of losartan on basal aldosterone production by rat AoSMC. Rat AoSMC were incubated with or without 10^−5^ M losartan for 2 h, and then the culture media had their aldosterone concentrations determined, as described in the text. Data are presented as the mean of three different experiments. Each experiment was performed in triplicate. ^a^ *p* < 0.001 *vs.* control of LETO, * *p* < 0.001 *vs.* control of OLETF (none).

The OLETF AoSMC culture medium displayed significantly greater (4–5 fold higher) basal aldosterone production than the LETO AoSMC culture medium (Figure 3). When the AoSMC were incubated with various concentrations of AII, the aldosterone production of the LETO AoSMC gradually increased, while the OLETF AoSMC did not display any increase in their aldosterone production (Figure 3). Losartan inhibited the basal aldosterone production of OLETF AoSMC, but not that of LETO AoSMC (Figure 4).

#### 2.3.2. Effects of HDL

**Figure 5:**
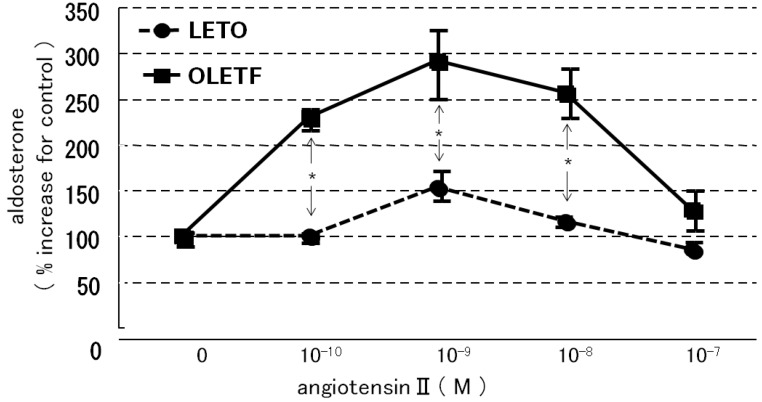
Effect of angiotensin II in the presence of 20 mg cholesterol/dL of HDL on aldosterone production by rat AoSMC. Data are expressed as percentage increases compared with the control. Data are presented as mean ± S.E.M. values of three different experiments. Each experiment was performed in triplicate, and aldosterone concentrations were determined using a radioimmunoassay. * *p* < 0.001 *vs.* LETO.

When the AoSMC were co-incubated in the presence of HDL, which supplied cholesterol as a substrate for steroidogenesis in rats, AII significantly increased greater Aldo production in the OLETF AoSMC than the LETO AoSMC, and Aldo production was maximally enhanced by 10^−9^ M angiotensin II (Figure 5).

### 2.4. AT1 Receptor Protein Expression

#### 2.4.1. Effect of Angiotensin II

**Figure 6:**
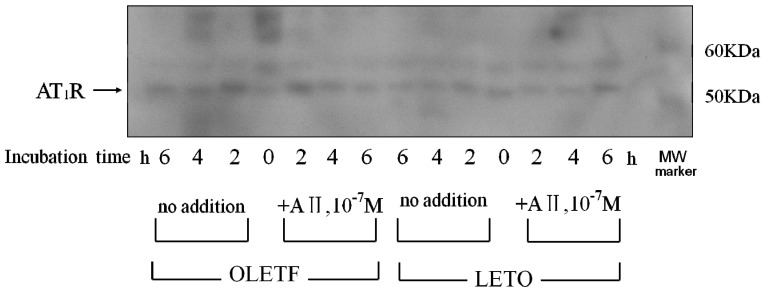
Effect of angiotensin II on AT1R protein expression. Rat AoSMC were incubated with “(+)” or without “(−)” 10^−7^ M angiotensin II for 2, 4 and 6 h. The pelleted cells were soon solubilized and subjected to SDS-PAGE using a linear gradient of 10%–20% acrylamide gel. All proteins were electrophoretically transferred to nitrocellulose membranes and analyzed using standard immunoblotting procedures. Molecular weight (MW) markers were run in the right lane.

The incubation of AoSMC without AII for 2, 4 and 6 h resulted in a much more marked increase in AT1R protein expression in the OLETF AoSMC than in the LETO AoSMC (Figure 6). When the LETO AoSMC were incubated with AII for 2 and 4 h, the AT1R protein expression was suppressed, comparing with that in the LETO AoSMC incubated without AII for 2 and 4 h (Figure 6). Moreover, the AT1R protein expression in the LETO AoSMC after stimulation with AII for 6 h was recovered up-to that after stimulation without AII for 6 h. On the contrary, 2 h’ incubation with AII did not suppress the AT1R protein expression of OLETF AoSMC (Figure 6), while 4 or 6 h’ incubation with AII relatively suppressed to the control level of the AT1R protein expression of OLETF AoSMC.

#### 2.4.2. Effect of Angiotensin I

**Figure 7:**
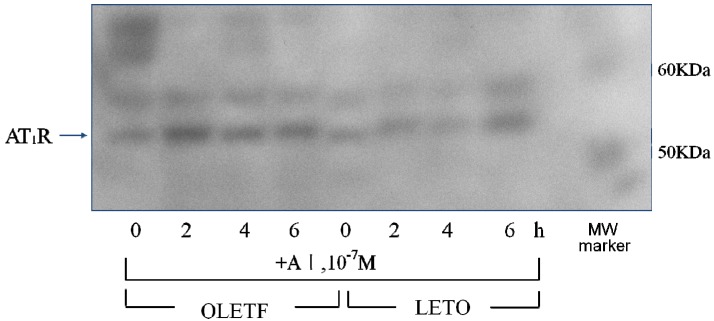
Effect of angiotensin I on AT1R protein expression.Rat AoSMC were incubated with 10^−7^ M angiotensin I for 2, 4 and 6 h. The pelleted cells were soon solubilized and subjected to SDS-PAGE using a linear gradient of 10%–20% acrylamide gel. All proteins were electrophoretically transferred to nitrocellulose membranes and analyzed using standard immunoblotting procedures. Molecular weight (MW) markers were run in the right lane.

The incubation of AoSMC with angiotensin I (AI) for 2, 4 and 6 h resulted in a much more marked increase in AT1R protein expression in the OLETF AoSMC than in the LETO AoSMC (Figure 7).

### 2.5. Discussion

We confirmed that cultured rat AoSMC expressed CYP11B2 mRNA and were able to produce aldosterone. Our findings are consistent with the previous findings obtained in SHRSP (spontaneously hypertensive rats-stroke prone) rats by Takeda [1]. Pregnenolone that had been produced by the rat AoSMC was also detected in the culture medium using a specific radioimmunoassay, and (Bu)2cAMP enhanced the pregnenolone production of the AoSMC, as is observed in the adrenal cortex. Therefore, it is suggested that cultured rat AoSMC possess steroidogenic activity; *i.e.*, they are able to produce pregnenolone and then use it to synthesize aldosterone as occurs in the adrenal glands. However, we do not know enough about the physiological or pathological role of the aldosterone produced in SMC. On the other hand, it is generally accepted that aldosterone can exert direct effects on the vascular system. For example, it is associated with fatal cardiovascular complications as it induces oxidative stress, inflammation, hypertrophic remodeling, fibrosis, and endothelial dysfunction [9]. Moreover, previous studies clearly demonstrated that thoracic aorta medial wall area containing collagen, significantly increased according to the aging in OLETF than in LETO when comparing that in 5-week old with 15- or 30-week old rats [14]. Thus, we can speculate the role of local excess aldosterone production in young OLETF rats at pre-diabetic stage may induce later onset of vascular dysfunction, although we did not try to examine pathological analysis of vasculatures in those model rats.

The results of the present study clearly demonstrated that AoSMC prepared from young OLETF rats produced higher basal levels of aldosterone compared with those from the control LETO rats. Thus, future onset of diabetic vascular dysfunction might be partly caused by excess aldosterone production by AoSMC. Moreover, it is suggested that the activity of the renin-angiotensin system is upregulated under diabetic conditions since losartan significantly suppressed aldosterone production by the AoSMC from the OLETF rats. The present data suggested that the OLETF AoSMC rather than the LETO AoSMC can produce angiotensin II. As shown in Figure 6 by qualitative analysis of AT1R, 2, 4 and 6 h’ incubation without AII caused a much more marked increase in AT1R protein expression in the AoSMC OLETF than in the LETO AoSMC, although we need furthermore experiments clarifying quantitatively changes in AT1R protein and mRNA expression regulated by various additives including AII in the OLETF and LETO AoSMC in future. Thus, it is suggested that the AT1R protein expression of the OLETF AoSMC is upregulated by AII itself. Conversely, incubation with AII for 2 and 4 h suppressed the AT1R protein expression of the LETO AoSMC, possibly via a negative feedback system, of which regulation was also observed in the OLETF AoSMC after stimulation with AII for 4 and 6 h. The present data also demonstrated that the AT1R protein of the OLETF AoSMC was much higher expressed than that of the LETO AoSMC, when incubated with A I for 2, 4 and 6 h (Figure 7). On the other hand, it was reported that human AoSMC possesses angiotensin converting enzyme (ACE), of which activity can be elevated by pulsatile mechanical pressure [15]. Moreover, the *in vitro* incubation study with high glucose, a diabetic-like condition, clearly demonstrated an increase in AII generation in rat AoSMC [16]. Thus, it is suggested that the OLETF AoSMC could produce higher amount of AII than the LETO AoSMC when incubated with A I for 2 h, possibly resulting in up-regulating the AT1R protein expression in the OLETF AoSMC.

It was reported that diabetic dyslipidemia and the modification of circulating lipoproteins promote adrenocortical aldosterone synthesis [17]. In addition, it was found that native HDL and modified HDL required Janus kinase-2 to allow them to combat increases in the demand for steroidogenesis [17]. Another study demonstrated that the cholesteryl esters in HDL are used as steroidogenic substrates in the rat adrenal cortex [18]. We previously reported that the activities of both acid and alkaline cholesterol esterase, which play a crucial role in regulating the cholesterol supply for steroidogenesis, were also increased by a substrate containing apo-HDL plus cholesteryl oleate and phosphatidylcholine more than by a substrate containing cholesteryl oleate plus apo-LDL with phosphatidylcholine or cholesteryl oleate with phosphatidylcholine [19]. Moreover, Simpson had already reported that in short-term incubations of fresh adrenal tissue, the supply of cholesterol may be a limiting factor in aldosterone synthesis and that HDL rather than LDL is the preferred source [20]. Rainey had also reported that HDL, especially HDL2, stimulated aldosterone synthesis by increasing expression of CYP11B2 [21]. These findings suggest that HDL play an important role in supplying cholesterol for adrenal steroidogenesis, including for aldosterone production. In the present study, concomitant stimulation with HDL and AII resulted in markedly enhanced aldosterone production in the OLETF AoSMC, suggesting that AII-induced aldosterone production is greatly enhanced by HDL, which induces steroidogenesis in rats [18,19], as is the case for LDL in humans. In basal conditions, the OLETF AoSMC synthesize greater amounts of aldosterone than the LETO AoSMC via activation of the AII-AT1R axis, which can be inhibited by losartan. Moreover, AII had more marked effects on aldosterone production in the OLETF AoSMC than in the LETO AoSMC when they were co-incubated with HDL, suggesting that AII induces the OLETF AoSMC, rather than the LETO AoSMC to synthesize aldosterone when sufficient amounts of HDL (substrates for aldosterone synthesis) are present. Moreover, it is suggested that endogenous cholesterol for aldosterone synthesis may be much more depleted in the OLETF AoSMC than the LETO AoSMC because of consumption of cholesterol for highly activated steroidogenic activity of basal aldosterone synthesis.

## 3. Experimental

### 3.1. Materials

All of the materials were purchased from Sigma Chemical Co. (St. Louis, MO, USA), unless otherwise stated. Culture medium consisting of Dulbecco’s modified Eagles’ medium supplemented with nutrient mixture F-12 (DMEM-F12) and fetal bovine serum (FBS) were obtained from Gibco, Tokyo, Japan, and San-Ko Pure Chemicals Co. (Tokyo, Japan), respectively. The angiotensin II receptor type I (AT1R) competitive antagonist losartan-DuP753 (2-*n*-butyl-4-chloro-5-hydroxymethyl-1- [2-(1*H*-tetrazole-5-yl)biphenyl-4-hy]methyl) imidazole; losartan) was kindly provided by Merck & Co., Inc. (West Point, PA, USA). A rabbit anti-peptide antibody against the rat AT1R was purchased from Chemicon International Inc. (Temecula, CA, USA, Lot # 19070265). Sodium dodecyl sulfate-polyacrylamide gel electrophoresis (SDS-PAGE) was performed using a linear gradient of 10%–20% acrylamide gel, which was purchased from Daiichi-Kagaku-Yakuhin (Tokyo, Japan). Human high-density lipoproteins (HDL) were prepared from healthy human serum, as described previously [22].

### 3.2. Animals

We subjected 6-week-old male Long-Evans Tokushima Otsuka rats (LETO: the control group) and Otsuka Long-Evans Tokushima Fatty rats (OLETF: the type 2 diabetes group), which were kindly supplied by Otsuka Pharmaceutical Co. (Tokushima, Japan) for the present experiments. After sacrificing the rats by decapitation, we collected their thoracic and abdominal aortae, and the AoSMC layer was dissected from the aortic outer layer.

### 3.3. Preparation of Rat Aortic Smooth Muscle Cells

Rat AoSMC were isolated from rat thoracic and abdominal aortae using the media explant method, before being cultured over several passages according to the method described by Ross [23]. The cells were cultured with DMEM-F12 containing 10% FBS, 100 units/mL penicillin (Gibco), and 100 μg/mL streptomycin (Gibco). The cells derived from the explants became relatively confluent within a period of approximately 2 weeks. They were then rinsed with phosphate buffered saline (PBS, Sigma), before being trypsinized by incubating them in a solution of 0.125% trypsin and 0.02% ethylenediaminetetraacetic acid (EDTA) in PBS for 1–2 min at 37 °C. The resultant cell suspensions were pipetted into 75-cm^2^ tissue culture flasks containing 10 mL culture medium and incubated. The experiments were performed using cells from passages 3–5.

### 3.4. Cell Number Determination

Cell number was determined using the CyQUANT cell proliferation assay kit (Molecular Probes Inc., Eugene, OR, USA), as reported previously [5].

### 3.5. Reverse Transcription and the Polymerase Chain Reaction

Total RNA was extracted and purified using the RNeasy Mini Kit (QIAGEN KK, Tokyo, Japan). Reverse transcription was carried out using 250 ng of total RNA annealed with oligo (dT) primers in a 20 μL reaction volume and a GeneAmp® RNA polymerase chain reaction (PCR) kit (Applied Biosystems, Foster City, CA, USA). The subsequent PCR was performed using the cDNA templates obtained from the RT reactions, 15 μM of the primer pairs, prepared as previously reported [10] and a GeneAmp 9600 PCR system (Applied Biosystems, Funakoshi-Yakuhin, Tokyo, Japan). The PCR conditions employed were as follows: initial denaturation at 95 °C for 105 s, followed by 35 cycles of 15 s at 95° C, 30 s at 60° C, and 7 min at 72 °C. The PCR products were run on agarose gels (2%) with ethidium bromide (50 ng/100 mL), as reported previously [5].

### 3.6. Pregnenolone Formation

The adherent rat AoSMC were seeded into 6-well culture plates at a concentration of 5 × 10^5^ cells/well in 2 mL of Krebs–Ringer bicarbonate (KRB) buffer (20 mM 4-(2-hydroxyethyl)-1-piperazineethanesulfonic acid (HEPES) [pH 7.4], 1.3 mM CaCl_2_, 0.2% glucose, and 0.1% bovine serum albumin (BSA)) (KRB-HEPES). The cells were preincubated in the presence of 30 μM trilostane, a specific inhibitor of 3β-hydroxysteroid dehydrogenase (HSD), for 15 min, before being incubated with various substances including HDL and angiotensin II (AII) for 2 h. After the abovementioned incubation procedure, the medium was collected, and its pregnenolone concentration was directly determined using a specific radioimmunoassay [5]. The viability of the cells, which was determined by trypan blue exclusion, was always greater than 94%.

### 3.7. Aldosterone Production

Rat AoSMC were harvested and incubated in the absence of trilostane in KRB-HEPES without BSA. The levels of aldosterone in the incubation media were measured with a specific radioimmunoassay kit (SPAC RIA Kit, Daiichi Radio-isotope Co., Tokyo, Japan). The sensitivity of the radioimmunoassay was 6.9 fmol/tube. The overall recovery rate of the radioimmunoassay was 88%–94%, and the inter-assay and intra-assay variation values were 8.2% and 6.5%, respectively.

### 3.8. Immunoblotting of the Angiotensin II Receptor

Rat AoSMC were preincubated with or without various inhibitors for 15 min in KRB-HEPES. After the preincubation step, AII was added to the culture media, which were then incubated for 2 h. Next, the culture media were centrifuged, and the resultant pellets were collected. The pelleted cells were soon solubilized in Seprazol and subjected to SDS-PAGE using a linear gradient of 10%–20% acrylamide gel. All proteins were electrophoretically transferred to nitrocellulose membranes (Amersham, Tokyo, Japan) and analyzed using the standard immunoblotting procedure. The AT1R protein was immunospecifically detected using a rabbit antipeptide antibody as the primary label and a goat anti-rabbit immunoglobulin G labeled with [^125^I] as the secondary label, and the resultant signals were detected using an autoradiogram.

### 3.9. Statistical Analysis

Results are expressed as the mean ± S.E.M. of at least three different experiments/measurements. We used four different rat AoSMC batches. Representative results from two batches are shown in the figures. The Student’s *t*-test was used for comparisons. Differences were considered significant when the associated *p*-value was less than 0.05.

## 4. Conclusions

To elucidate the mechanism responsible for vascular aldosterone synthesis in various pathological conditions, including diabetes, we examined the ability of AoSMC prepared from spontaneously diabetic rats to produce aldosterone and the regulatory mechanism that controls their aldosterone production. CYP11B2 mRNA expression was detected in both the LETO and OLETF AoSMC. Basal aldosterone production was significantly greater in the OLETF AoSMC culture medium than in the LETO AoSMC culture medium. Thus, it is suggested that an enhancement in aldosterone production of the vascular wall in OLETF even at the pre-diabetic stage, may possibly explain future onset of diabetic vascular dysfunction.

## References

[1] Takeda Y. (2004). Vascular synthesis of aldosterone: Role in hypertension. Mol. Cell. Endocrinol..

[2] Takeda Y., Miyamori I., Inaba S., Furukawa K., Hatakeyama H., Yoneda T., Mabuchi H., Takeda R. (1997). Vascular aldosterone in genetically hypertensive rats. Hypertension.

[3] Hatakeyama H., Miyamori I., Takeda Y., Yamamoto H., Mabuchi H. (1996). The expression of steroidogenic enzyme genes in human vascular cells. Biochem. Mol. Biol. Int..

[4] Silvestre J.S., Robert V., Heymes C., Aupetit-Faisant B., Mouas C., Moalic J.M., Swynghedauw B., Delcayre C. (1998). Myocardial production of aldosterone and corticosterone in the rat. Physiological regulation. J. Biol. Chem..

[5] Nishikawa T., Suematsu S., Saito J., Soyama A., Ito H., Kono T., Chrousos G. (2005). Human renal mesangial cells produce aldosterone in response to low-density lipoprotein (LDL). J. Steroid Biochem. Mol. Biol..

[6] Siragy H.M., Xue C. (2008). Local renal aldosterone production induces inflammation and matrix formation in kidneys of diabetic rats. Exp. Physiol..

[7] Gomez-Sanchez C.E., Zhou M.Y., Cozza E.N., Morita H., Foecking M.F., Gomez-Sanchez E.P. (1997). Aldosterone biosynthesis in the rat brain. Endocrinology.

[8] Bender S.B., McGraw A.P., Jaffe I.Z., Sowers J.R. (2013). Mineralocorticoid receptor-mediated vascular insulin resistance: An early contributor to diabetes-related vascular disease?. Diabetes.

[9] Briet M., Schiffrin E.L. (2013). Vascular actions of aldosterone. J. Vasc. Res..

[10] Nishikawa T., Matsuzawa Y., Suematsu S., Saito J., Omura M., Kino T. (2010). Effect of atorvastatin on aldsterone production induced by glucose, LDL or angiotensin IIin human renal mesangial cells. Arzneim. Forsch..

[11] Hostetter T.H., Rosenberg M.E., Ibrahim H.N., Juknevicius I. (2001). Aldosterone in renal disease. Curr. Opin. Nephrol. Hypertens..

[12] Kawano K., Hirashima T., Mori S., Natori T. (1994). OLETF (Otsuka Long-Evans Tokushima Fatty) rat: A new NIDDM rat strain. Diabetes Res. Clin. Pract..

[13] Nemoto S., Taguchi K., Matsumoto T., Kamata K., Kobayashi T. (2012). Pravastatin normalizes ET-1-induced contraction in the aorta of type 2 diabetic OLETF rats by suppressing the KSR1/ERK complex. Am. J. Physiol. Heart Circ. Physiol..

[14] Hosomi. H., Noma T., Ohyama H., Takahashi T., Kohno M. (2002). Vascular proliferation and transforming growth factor-β expression in pre-and early stage of diabetes mellitus in Otsuka Long-Evans Tokushima fatty rats. Atherosclerosis.

[15] Iizuka K., Machida T., Kawaguchi H., Hirafuji M. (2008). Pulsatile mechanical pressure promotes angiotensin-converting enzyme expression in aortic smooth muscle cells. Cardiovasc. Drugs Ther..

[16] Lavrentyev E.N., Estes A.M., Malik K.U. (2007). Mechanism of high glucose induced angiotensin II production in rat vascular smooth muscle cells. Circ. Res..

[17] Saha S., Willenberg H.S., Bornstein S.R., Graessler J., Kopprasch S. (2012). Diabetic lipoproteins and adrenal aldosterone synthesis—A possible pathophysiological link?. Horm. Metab. Res..

[18] Gwynne J.T., Mahaffee D.D. (1989). Rat adrenal uptake and metabolism of high density lipoprotein cholesteryl ester. J. Biol. Chem..

[19] Nishikawa T., Mikami K., Yoshida A., Omura M., Tamura Y., Saito Y. (1993). Regulation of cholesterol metabolism in adrenal cortex: Effects of apoproteins on cholesterol esterase in rat adrenal glands. Endocr. J..

[20] Simpson H.D., Shepherd R., Shepherd J., Fraser R., Lever A.F., Kenyon C.J. (1989). Effects of cholesterol and lipoproteins on aldosterone secretion by bovine zona glomerulosa cells. J. Endocrinol..

[21] Xing Y., Cohen A., Rothblat G., Sankaranarayanan S., Weibel G., Royer L., Francone O.L., Rainey W.E. (2011). Aldosterone production in human adrenocortical cells is stimulated by high-density lipoprotein 2 (HDL2) through increased expression of aldosterone synthase (CYP11B2). Endocrinology.

[22] Yaguchi H., Tsutsumi K., Shimono K., Omura M., Sasano H., Nishikawa T. (1998). Involvement of high density lipoprotein as substrate cholestero1 for steroidogenesis by bovine adrenal fasciculo-reticu1aris cel1s. Life Sci..

[23] Ross R. (1971). Growth of smooth muscle in culture and formation of elastic fibers. J. Cell Biol..

